# Measurement of body composition by deuterium oxide dilution technique and development of a predictive equation for body fat mass among severe neurologically impaired children

**DOI:** 10.3389/fnut.2023.1162956

**Published:** 2023-10-18

**Authors:** Wirada Sakamornchai, Oraporn Dumrongwongsiri, Jeeraparn Phosuwattanakul, Sirinapa Siwarom

**Affiliations:** ^1^Division of Nutrition, Department of Pediatrics, Faculty of Medicine Ramathibodi Hospital, Mahidol University, Bangkok, Thailand; ^2^Department of Pediatrics, Faculty of Medicine Chiang Mai University, Chiang Mai, Thailand

**Keywords:** deuterium oxide dilution technique, body composition, anthropometric measurement, neurologically impaired children, home enteral feeding

## Abstract

**Introduction:**

Neurologically impaired (NI) children are at risk of malnutrition, which consequently impacts their health and quality of life. Accurate nutrition assessment is an important step in guiding appropriate nutrition support. Conventional anthropometric measurements among NI children have some limitations. Determining body composition requires more complex equipment, which is not routinely performed. This study was conducted to evaluate the association between anthropometric parameters and body composition assessed using the deuterium dilution technique (DDT) in NI children.

**Methods:**

A cross-sectional study enrolled severe NI children aged 1–20 years who received home enteral nutrition for at least 3 months. Weight, length, and 4-site skinfold thickness were measured. Body composition was determined using DDT following the International Atomic Energy Agency (IAEA) protocol.

**Results:**

A total of 37 NI children (56.76% male, median age 7.2 years) were enrolled. The prevalence of underweight, stunting, and overweight were 22, 38, and 35%, respectively. Body composition analysis showed the mean (SD) of total body water (TBW) and fat mass (FM) were 10.52 (4.51) kg and 9.51 (6.04) kg, respectively. Multivariate GLM analysis showed that the factors associated with FM were age (β = 0.07 [0.05,0.08]; *p* < 0.001), body mass index (BMI) (β = 0.82 [0.52, 1.12]; *p* < 0.001), biceps skinfold thickness (BSF) (β = 0.49 [0.23,0.75]; *p* = 0.001), and subscapular skinfold thickness (SSF) (β = −0.24 [−0.46,0.03]; *p* = 0.030). A predictive equation for FM was constructed.

**Conclusion:**

A high prevalence of malnutrition was found among severe NI children despite enteral nutrition support. Our findings showed that age, BMI, BSF, and SSF were associated with FM. The predictive equation of FM was proposed and needed to be further validated and applied to clinical practice.

## Introduction

Neurological impairment (NI) in children affects their development and cognitive function, which also impacts oral motor function and feeding development (1). Malnutrition is commonly found in children with NI, with a reported prevalence of 46–90% from previous studies (2–4). Malnutrition causes muscle weakness, impaired immune function, and prolonged hospitalization in children with NI. Several factors, including the types and severity of underlying diseases, ambulatory and cognitive status, and medication use, contribute to the risk of malnutrition (5). In addition, 90% of NI children are affected by gastrointestinal disorders such as oropharyngeal dysphagia and gastroesophageal reflux disease (GERD) (5), which are associated with inadequate diet intake and respiratory complications. Some children with severe NI need enteral nutrition support due to inadequate nutrient intake via oral route or having contraindications to oral feeding (3). Overweight and obesity may be found in NI children with enteral nutrition support (6).

Accurate nutrition assessment, including medical and dietary history, anthropometric measurements and growth assessment, physical examination, and specific investigations, is essential for providing appropriate nutrition support to NI children. Frequently, routine anthropometric measurements such as weight and height/length are inaccurately assessed in NI children due to limitations such as bedridden status, joint contractions, and deformities. In addition, growth assessment of NI children is difficult because of the limited information on the disease-specific growth curve. A single anthropometric parameter such as body mass index (BMI), weight, or length is not an optimal indicator of nutrition status among NI children (7–9). Body composition is also an essential parameter for monitoring nutrition status, but the assessment is more complicated. NI children have a different proportion of fat and muscle than normal children, depending on their underlying diseases and severity. Consequently, some proposed estimations of fat mass (FM) or fat-free mass (FFM) from anthropometric parameters in normal children could not accurately determine the body composition of NI children. Measurement of body composition among NI children using standard techniques, including whole-body dual-energy X-ray absorptiometry (DXA), bioelectrical impedance analysis (BIA), and isotope dilution technique, has some limitations. DXA is costly and requires more complex equipment. The isotope dilution technique provides accurate information but is usually performed in research rather than clinical practice due to its sophisticated processes and cost. BIAs are widely used in clinical practice, but more advanced equipment is needed when performing the measurement in NI children who cannot stand or cooperate with the assessment process. The equipment for BIA measurements in the supine position is not available in some areas.

In Thailand, information regarding nutrition status and management in NI children is scarce. Many NI children suffer from malnutrition and feeding problems, and some of them need enteral nutrition support. Nutrition assessment is comprehensively performed in NI children, but there are some limitations to demonstrating their accurate nutrition status. Body composition could not be routinely assessed by the standard method due to limited resources. Therefore, this study aimed to investigate the association between anthropometric parameters and the body composition of children with severe NI who depended on enteral nutrition support. We also aimed to develop a predictive equation that may help estimate body composition based on the bedside anthropometric parameters.

## Materials and methods

A cross-sectional study was performed from June 2021 to March 2022 at the Nutrition Clinic, Department of Pediatrics, Faculty of Medicine, Ramathibodi Hospital, Bangkok, Thailand. NI children, aged 1–20 years, with home enteral nutrition support for at least 3 months were enrolled. Children who had abnormal hydration status (either dehydration or edema, by history and physical examination), acute illness, or active infection, and who were unable to be fed on sampling day, were excluded from this study. The study protocol was approved by the Human Research Ethics Committee, Faculty of Medicine, Ramathibodi Hospital, Mahidol University (Protocol No. COA. MURA2021/390). The detailed study protocol was explained to parents or legal guardians, and informed consent was obtained before enrollment. The study was performed in accordance with the Declaration of Helsinki.

In this study, we used the deuterium oxide dilution technique (DDT) to determine body composition in NI children. The sample size for a single mean estimation was calculated by STATA version 16.1. Following the standard method described by Rieken et al. (10), the mean (SD) of total body water (TBW) determined using DDT in NI children with gastrostomy tubes, which was 10.3 (3.8) kg, was used to estimate the sample size in this study. With a level of confidence of 95% and a power of 80%, the calculated sample size for our study was 45.

### Baseline characteristics

Baseline characteristics, including age, sex, underlying neurological diseases, presence of seizures, and feeding methods (enteral feeding with/without oral route), were reviewed from the medical record. The severity of NI was assessed by motor mobility using the Gross Motor Functional Classification System (GMFCS), from the less severe or ability to walk without restrictions (level I) to the most severe or limited voluntary movement and the ability to maintain antigravity in the head and trunk (level V) (11). The characteristics of NI were classified as spasticity, dyskinesia, and hypotonia by researchers or pediatric neurologists. Pubertal development was assessed using Tanner staging (Tanner stages I–V) by the researchers.

### Anthropometric assessments

Anthropometric measurements, including weight, length, mid-upper arm circumference (MUAC), and skinfold thickness, were performed by the researchers on the day of data collection.

Body weight while wearing a light cloth or naked without a diaper was obtained using a standard digital scale to the nearest 0.1 kg. Children aged less than 24 months were weighed on a pan-type digital scale (Seca^®^ 374, Seca Corporation, Hamburg, Germany). Children aged older than 24 months who could stand without assistance were weighed on a platform digital scale (Seca^®^ 284, Seca Corporation, Hamburg, Germany), whereas those who were unable to stand were weighed by subtracting their parents’ weight after being weighed together while holding their child.

Length was measured to the nearest 0.1 cm using an infantometer (Seca^®^ 416, Seca Corporation, Hamburg, Germany). Because an infantometer is limited to 100 cm, the researchers used a self-made measuring instrument for children over 100 cm. The instrument was made with a 150 cm flexible, non-stretchable tape attached to a head and foot plastic board, routinely used in our nutrition clinic for length assessment of NI children. Length could not be accurately measured in some NI children with scoliosis or joint contraction. Instead, length was estimated from knee height by a predictive equation developed by Stevenson (12). Knee height was assessed using a flexible, non-stretchable tape that measured from heel to the anterior surface of the thigh, approximately 3 cm above the patella, with a 90 degree angle between thigh to leg and leg to foot (13).

MUAC was measured at the midway point between the uppermost edge of the posterior border of the acromion process and the olecranon process of both arms using a flexible, non-stretchable tape when the arm was hanging loosely. Skinfold thickness was measured to the nearest 0.1 mm at four different sites, i.e., biceps (BSF), triceps (TSF), subscapular (SSF), and suprailiac (SiSF) skinfold, both right and left sides, using Holtain caliper (Holtain Ltd., Crymych, United Kingdom), following the standard method described by Lee and Nieman (14).

All anthropometric parameters were assessed following the standard method by trained dietitians, nurses, and researchers. The measurements of length, knee height, MUAC, and skinfold thickness were repeated three times. The represented values were determined by the average of the three values from each measurement. Anthropometric parameters were calculated using Z-scores, including length-for-age Z-score (LAZ), weight-for-length Z-score (WLZ), weight-for-age Z-score (WAZ), BMI-for-age Z-score (BMIZ), MUAC-for-age Z-score, TSF-for-age Z-score, and SSF-for-age Z-score, were calculated using the WHO Anthro software (15) for children aged 0–60 months and the WHO AnthroPlus software (16) for children aged 5–19 years.

### Body composition assessment

Body composition was assessed using DDT. The principle of this technique is to assess TBW with deuterium oxide (D_2_O), and then calculate FFM from TBW. The DDT was performed according to the protocol proposed by the International Atomic Energy Agency (IAEA) (17). In brief, the appropriate amount of D_2_O, according to the participant’s weight, was fed via a feeding tube, followed by 10 mL of sterile water. D_2_O was traced from its concentration in saliva to its equilibrium. At least 2 mL of saliva was collected before D_2_O dosing, then repeated at 3 and 4 h post-dosing. The researchers collected saliva samples by putting a cotton swab into the mouth of each participant and extracted the saliva into microtubes using a syringe plugger. The samples were then kept frozen until analysis of D_2_O enrichment using Fourier transform infrared (FTIR) spectroscopy at the Institute of Nutrition Mahidol University (INMU). TBW was calculated using the information of the initial dose and enrichment of D_2_O at equilibrium. Once TBW was calculated, FFM was determined based on the value of TBW using the FFM hydration factors suggested in the IAEA protocol, which vary by age and sex (13). Then, FM was calculated by subtracting FFM from body weight.

In addition to DDT, FM was determined by predictive equations using the variables from bedside anthropometric measurements. We calculated body FM percentage based on the predictive equations proposed by previous studies and compared it with the value of body FM percentage obtained from DDT. These predictive equations were the following:

1) The Baton Rouge Children’s Study (18) suggested the equation to determine body FM percentage using the data of 4-site skinfold thickness as

$$\begin{array}{l} FM\;\left(\%\right)=8.71+0.19\;\left(SSF\right)+0.76\;\left(BSF\right)\\ \;+0.18\;\left(\mathrm{SiSF}\right)+0.33\;\left(TSF\right) \end{array}$$

2) Modified Slaughter equations, which were modified from the original Slaughter equations by additional correction factors for children with cerebral palsy (19).

### Statistical analysis

Statistical analysis was performed using STATA version 16.1 for Windows (StataCorp LLC, Texas, United States). Calculated probabilities (*p*-value) < 0.05 were considered statistically significant. Descriptive data were tested for normality using the Shapiro–Wilk test. Normal distributed data were presented as mean and standard deviation (SD), and non-normal distributed data were presented as median and interquartile range. A comparison of FM from predictive equations and DDT used paired *t*-tests and Bland–Altman analysis. Associations between body composition parameters (TBW and FM) from DDT and anthropometric parameters were analyzed using a univariate generalized linear model (GLM). Variables with significant associations (*p*-value <0.05) were assessed by multivariate GLM using backward elimination, log-likelihood, and likelihood ratio tests to obtain minimum variables that preserve the power of the model. The model was presented as a calibration plot of prediction model performance by adjusted R-square. The concordance correlation coefficient was analyzed by Lin’s approach (20). Testing of multicollinearity was performed with variance inflation factor (VIF) heteroskedasticity using the Breusch-Pagan/Cook-Weisberg test (21). If VIF was less than 5, the model was accepted (22).

## Results

Thirty-seven NI children were enrolled in this study. The baseline characteristics of participants are shown in Table 1. The median (IQR) age was 7.42 (3.67, 10.42) years. Spasticity was the most common type of NI. All participants were classified in GMFCS IV-V. All baseline characteristics were not different in the subgroup analysis, either by type or etiology of NI (asphyxia vs. non-asphyxia).

**Table 1:** Baseline characteristics of study participants (*n* = 37).

Characteristics	Number (%)
Sex, *n* (%)	
Male	21 (56.76)
Female	16 (43.24)
Sexual maturation, *n* (%)	
Prepuberty	26 (70.27)
Puberty	11 (29.73)
Types of NI, *n* (%)	
Dyskinesia	6 (16.22)
Hypotonia	6 (16.22)
Spasticity	25 (67.57)
Etiology of NI, *n* (%)	
Asphyxia	9 (24.32)
Brain malformation	6 (16.22)
CNS infection	1 (2.70)
Epilepsy	2 (5.41)
Genetic disease	9 (24.32)
Inflammation	1 (2.70)
Stroke	6 (16.22)
Toxic or metabolic disease	3 (8.11)
Severity, *n* (%)	
GMFCS IV-V	37 (100)
Presence of seizure, *n* (%)	
No	16 (43.24)
Yes	21 (56.76)
Oral intake, *n* (%)	
No	36 (97.30)
Yes	1 (2.70)

NI, neurologically impairment; GMFCS, gross motor functional classification system.

### Anthropometric assessments and body composition

Anthropometric parameters are presented in Table 2. The proportions of participants with underweight (WAZ < -2), stunting (LAZ < -2), and wasting (BMIZ <-2) were 22, 38, and 22%, respectively. Meanwhile, 35% of participants were overweight (BMIZ >2). The mean (SD) of FM and TBW from DDT were 9.51 kg (6.04) and 10.52 kg (4.51), respectively. Anthropometric parameters and body composition were not different between types or etiologies of NI.

**Table 2:** Anthropometric parameters of study participants.

Parameters	Represented value
Weight-for-age Z-score; WAZ (*n* = 27)^a^	
WAZ, median (IQR)	−0.73 (−1.83, 1.08)
WAZ classification, *n* (%)	
<−2	6 (22.22)
−2 to 2	19 (70.37)
>2	2 (7.41)
Length-for-age Z-score; LAZ (*n* = 37)	
LAZ, median (IQR)	−1.79 (−3.23, −0.59)
LAZ classification, *n* (%)	
<−2	14 (37.84)
≥−2	23 (62.16)
BMI Z-score; BMIZ (*n* = 37)	
BMIZ, median (IQR)	0.36 (−1.08, 2.06)
BMIZ classification, *n* (%)	
<−2	8 (21.62)
−2 to 2	16 (43.24)
>2	13 (35.14)
Mid-upper arm circumference (MUAC) Z-score (*n* = 13)^b^	
MUAC Z-score, mean (SD)	0.99 (2.57)
MUAC Z-score, *n* (%)	
<−2	3 (23.08)
≥−2	10 (76.92)
Triceps skinfold thickness (TSF) Z-score (*n* = 13)^b^	
TSF Z-score, mean (SD)	2.98 (1.68)
Subscapular skinfold thickness (SSF) Z-score (*n* = 13)^b^	
SSF Z-score, mean (SD)	3.70 (2.94)

a WAZ was assessed in children aged under 10 years.

b MUAC, TSF, and SSF Z-score were assessed in children aged under 5 years. BMI, body mass index.

**Figure 1:**
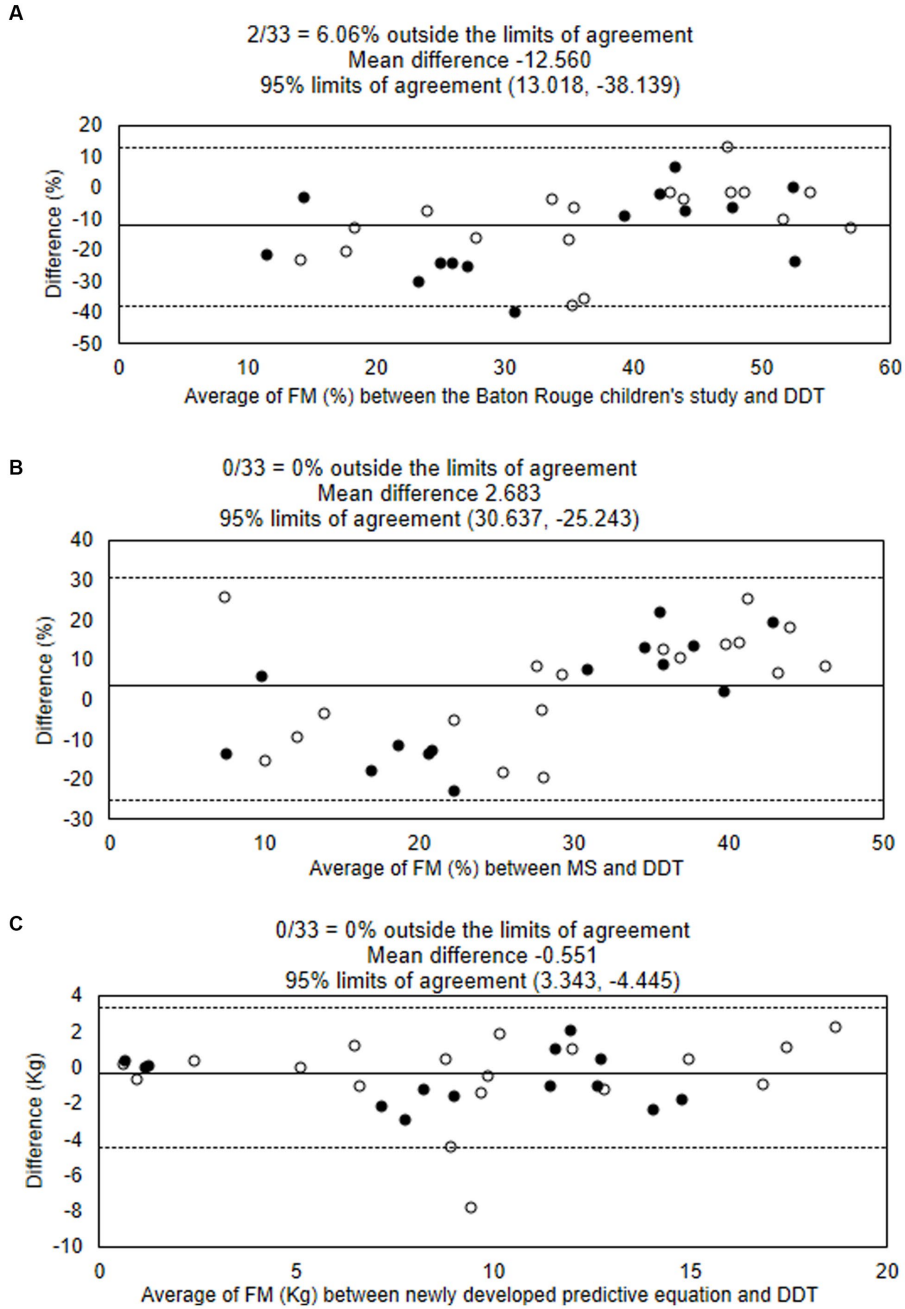
Bland–Altman analysis showing the agreement of body fat mass percentages estimated using the deuterium oxide dilution technique (DDT), the Baton Rouge study’s equation **(A)**, and Modified Slaughter equation **(B)**. The agreement of fat mass (kg) estimated using DDT and the newly developed equation is shown **(C)**. Solid lines show means, and dotted lines show limits of agreement. ○ male ● female. DDT, deuterium oxide dilution technique; FM, fat mass; MS, Modified Slaughter equation.

Body FM percentage obtained from DDT was significantly lower than that estimated by Baton’s equation (mean difference − 12.72 [−17.24, −8.20]; *p* < 0.001), but tended to be higher than that estimated by modified Slaughter equation without statistical significance (mean difference 3.02 [−2.00, 8.04]; *p* = 0.229), as shown in Table 3. The Bland–Altman analysis showing the agreement of body FM percentage calculated from previous predictive equations and DDT is presented in Figures 1A,B. The body FM percentage calculated using DDT was 13% lower than that calculated using Baton’s equation. While comparing with the modified Slaughter equation, the analysis showed a wide limit of agreement. Body FM percentage tended to have a negative bias toward small values but a positive bias when the mean body FM percentage using both methods was over 30%.

**Table 3:** Comparison of body fat mass percentage estimated using deuterium dilution technique (DDT) and predictive equations.

Estimation methods	Body fat mass percentage mean (SD)	Mean difference (95% CI)	*p*-value
The Baton Rouge Children’s study (18)	43.10 (12.28)	−12.72 (−17.24, −8.20)	<0.001
DDT	30.38 (17.34)		
Modified Slaughter equation (19)	27.34 (9.96)	3.02 (−2.00, 8.04)	0.229
DDT	30.38 (17.34)		

### Association between anthropometric parameters and body composition

The linear relationships between FM and TBW with participants’ age and anthropometric parameters evaluated using univariate GLM analysis are shown in Table 4. The variables with significant associations were included in the multivariate GLM analysis. The factors associated with FM from multivariate GLM analysis were age in months (β = 0.07 [0.05, 0.08]; *p* < 0.001), BMI (β = 0.82 [0.52, 1.12]; *p* < 0.001), BSF (β = 0.49 [0.23, 0.75]; *p* = 0.001), and SSF (β = −0.24 [−0.46,0.03]; *p* < 0.030). From these associations, we constructed the predictive equation to estimate FM as.


$$\begin{array}{l} FM\;\left(\mathrm{kg}\right)=\left[0.07^{*}\mathrm{age}\;\left(\mathrm{months}\right)\right]+\left[0.82^{*}BMI\;\left(\mathrm{kg}/m^{2}\right)\right]\\ \;+\left[0.49^{*}BSF\;\left(\mathrm{mm}\right)\right]-\left[0.24^{*}SSF\;\left(\mathrm{mm}\right)\right]-13.84 \end{array}$$

**Table 4:** Univariate generalized linear model between body composition and variable parameters.

Variable parameters	FM (kg)		TBW (kg)	
	β (95% CI)	*p*-value	β (95% CI)	*p*-value
Age (months)	0.08 (0.05, 0.11)	<0.001	0.07 (0.06, 0.09)	<0.001
BMI (kg/m^2^)	1.39 (0.96, 1.82)	0.001	0.45 (−0.01, 0.91)	0.054
WAZ	1.43 (0.87, 1.97)	<0.001	0.62 (0.12, 1.12)	0.015
LAZ	0.00 (−0.68, 0.68)	0.993	0.00 (−0.51, 0.51)	0.998
WLZ	1.21 (0.85, 1.58)	<0.001	0.42 (0.25, 0.59)	<0.001
BMI z-score	1.34 (0.51, 2.17)	0.002	0.09 (−0.63, 0.80)	0.813
MUAC (mm)	1.15 (0.84, 1.46)	<0.001	0.65 (0.35, 0.96)	<0.001
MUAC z-score	1.23 (0.76, 1.70)	<0.001	0.47 (0.30, 0.63)	<0.001
BSF (mm)	0.67 (0.33, 1.00)	<0.001	0.12 (−0.19, 0.42)	0.451
TSF (mm)	0.66 (0.40, 0.92)	<0.001	0.15 (−0.10, 0.41)	0.234
TSF z-score	1.43 (0.41, 2,46)	0.006	0.50 (0.10, 0.90)	0.014
SSF (mm)	0.53 (0.25, 0.80)	<0.001	0.22 (−0.01, 0.46)	0.064
SSF z-score	1.30 (0.57, 2.04)	0.001	0.48 (0.20, 0.76)	0.001
SiSF (mm)	0.50 (0.25, 0.75)	<0.001	0.16 (−0.07, 0.38)	0.168

BMI, body mass index; BSF, biceps skinfold thickness; FM, fat mass; LAZ, length-for-age Z-score; MUAC, mid-upper arm circumference; SiSF, suprailiac skinfold thickness; SSF, subscapular skinfold thickness; TBW, total body water; TSF, triceps skinfold thickness; WAZ, weight-for-age Z-score.

**Figure 2:**
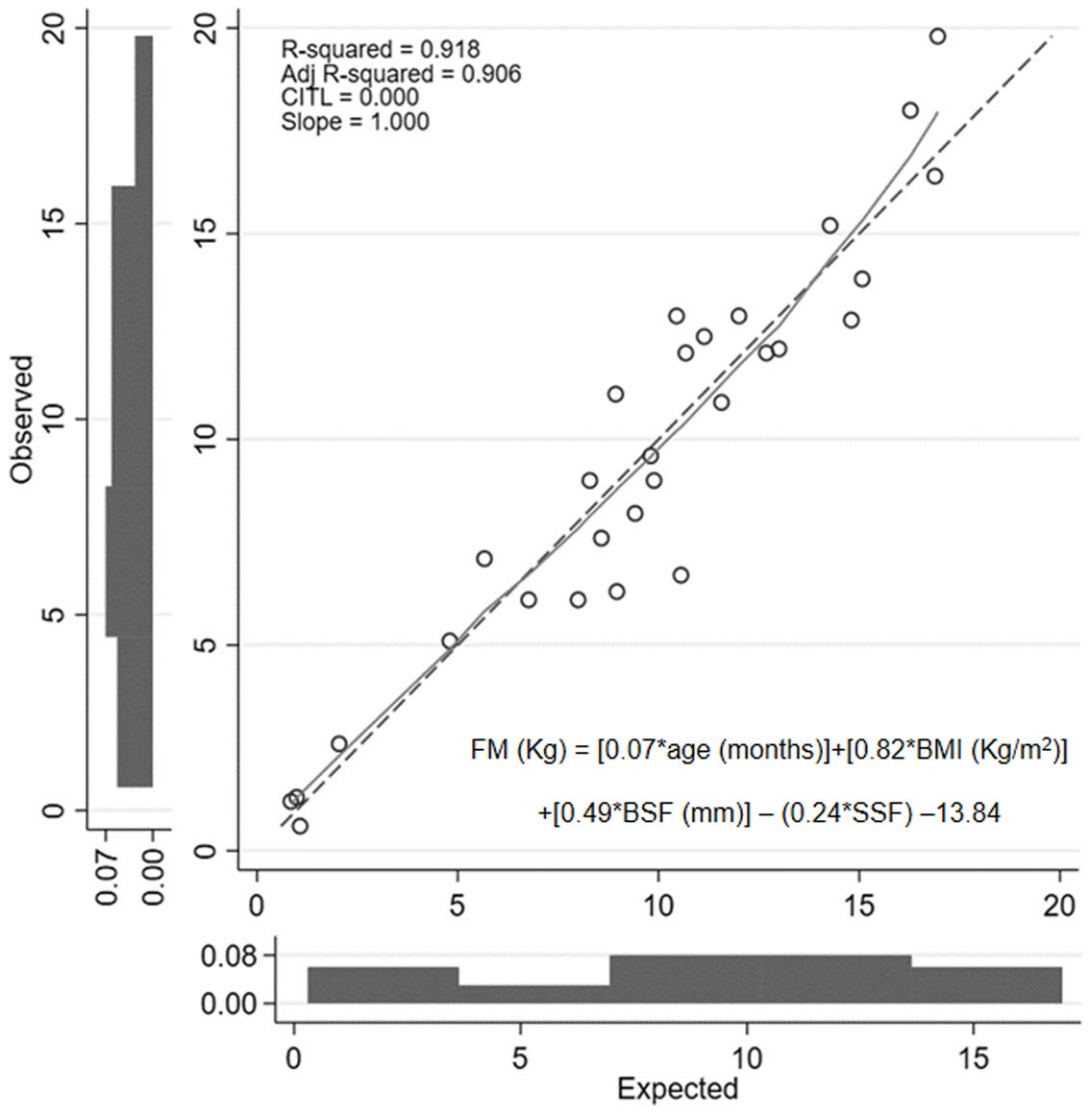
Calibration plot of fat mass value obtained from newly developed predictive equation compared with the value determined using the deuterium dilution technique. FM, fat mass; BMI, body mass index; BSF, biceps skinfold thickness; SSF, subscapular skinfold thickness.

The model analysis showed adjusted R^2^ = 0.906. Figure 2 shows the explanatory power of the predictive equation. Validation of the equation showed that factors in this newly developed equation had no multicollinearity; VIF for age, BMI, BSF, and SSF were 1.32, 2.41, 4.70, and 4.23, respectively. The concordance correlation coefficient was 0.984. This equation showed no heteroskedasticity (*p* = 0.090). The mean absolute percentage error (MAPE) was 23.53%, and the greater of two *p*-values from the one-side test for equivalent (TOST) was 0.009. The Bland–Altman analysis between FM estimated by the newly developed equation and DDT is presented in Figure 1C.

## Discussion

Our study presented a comprehensive assessment of the nutrition status of severe NI children with home-based nutritional support. Body composition was evaluated using DDT, and the association between anthropometric parameters and FM in NI children was demonstrated. Malnutrition, including wasting, stunting, and overweight, was found in more than half of the study participants.

Our study found a lower prevalence of undernutrition (wasting and stunting) and a higher prevalence of overweight among NI children compared to previous studies (2, 23). Our center provides a home-based nutrition program aiming to promote good nutrition status among children receiving home-based nutrition. Their feeding and growth are regularly monitored by nutritionists and pediatricians. Therefore, the study participants had received nutrition support and improved their nutrition status before study enrollment. However, one-third of the participants were overweight or obese. We found that it is difficult to determine the appropriate energy intake for children with severe NI as they have limited physical activity and might have a lower basal metabolic rate compared to children with normal development (24).

A previous study found that FM was varied by the age of children and severity. Our study also showed that the age of children was one of the factors associated with FM. The mean body FM percentage in our study populations was lower than the finding from a study by Rieken et al. (10), which enrolled NI children with a higher mean age compared to our study. FM is also affected by the severity of NI assessed by GMFCS and the presence of enteral feeding via gastrostomy tube (10, 19). In contrast, our study did not find this association due to the limited number of study participants and no variation in disease severity. While comparing with healthy children, we found that NI children had higher FM than healthy children (25, 26). Limited physical activity in NI children causes higher FM compared to healthy children. This is a challenging issue in monitoring nutrition status in NI children, as normal weight or BMI may reflect low muscle mass and high FM.

We compared the body FM percentage calculated from DDT and predictive equations that were previously developed (18, 19). Our result demonstrated an overestimation of FM percentage among children with NI by the equation developed from the data of healthy children (the Baton Rouge study) (18). In contrast, the body FM percentage from DDT was similar to the value obtained from the Slaughter equations with correction factors for NI children. A previous study showed an underestimation of FM percentage when using modified Slaughter equations compared to DXA among children with cerebral palsy, but the difference was lower among children with more severe disease (19). Another study found that children with cerebral palsy who had a similar FM percentage to healthy children assessed by DXA had a lower Z-score for anthropometric parameters (27). The authors hypothesized that NI children had relatively more internal fat than peripheral fat distribution. Using parameters of peripheral fat deposit, such as TSF, in a predictive equation could cause an underestimation of body FM percentage. A recent study reported a difference in glucose metabolism and insulin resistance between children with NI and healthy children (28), which may be due to different patterns of body fat distribution and physical activity. Our result showed a trend in overestimation of FM percentage by Baton Rouge and modified Slaughter equation among children with a higher FM percentage, as shown in the Bland–Altman plot (Figure 1). We assumed that fat distribution may be different between NI children with undernutrition and overweight, which needed to be explored in further study. Another factor influencing body FM percentage in children is the pubertal stage, and some studies integrated pubertal status into the predictive equations. Most of our study participants (70%) were in the pre-pubertal stage, and we could not find an association between FM parameters and pubertal status.

FM was recommended to be assessed as a part of nutrition monitoring in NI children (9); however, a single anthropometric parameter is a poor indicator of body FM. Kuperminc et al. (27) demonstrated that a single parameter, including BMI, MUAC, TSF, and upper arm fat area, had poor predictive ability to estimate FM among children with cerebral palsy. Many previous studies, both in healthy and NI children, used the multiple-site skinfold thickness as a predictor for FM. Rieken et al. (10) used the sum of four-site skinfold thickness measurements to predict FM among children with NI. A study in premenarcheal girls by Scerpella et al. (29) also showed that adding BSF to age, weight, and height parameters could improve the accuracy of FM prediction. The addition of TSF and SSF to a model containing BMI was found to reduce the prediction error by 20–30% compared to using BMI alone to predict FM in healthy children (30). Our findings showed that BMI, BSF, and SSF were associated with FM and were included in the proposed predictive equation.

The strength of this study is the assessment of the body composition of NI children receiving home enteral nutrition support by the gold standard technique, which reveals the association of body composition with bedside anthropometric measurements. The predictive model to determine FM in NI children was developed based on the findings of our study. The predictive model of FM may be useful, especially in resource-limited settings where the equipment for body composition assessment is not available. With the information about body composition, we can provide a more appropriate nutrition management plan and better nutrition support to NI children. Consequently, they can recover from malnutrition and improve their quality of life. However, the application of predictive models in clinical practice should be further evaluated. There are some limitations to this study. First, the determination of malnutrition was performed using criteria for children with normal development because the data for growth assessment in NI children is limited. Second, the number of participants was lower than the expected sample size. As the study was performed during the COVID-19 pandemic, some families of eligible NI children were unwilling to visit the hospital and spent 4–5 h to complete the study. In addition to the number of participants we were able to enroll, 4 of 37 saliva samplings were errors in the DDT analysis. The reduced number of participants from the number expected by the sample size calculation causes a reduction in study power from 80 to 65%.

This predictive equation for the determination of FM in NI children using anthropometric parameters and age is a simple equation that may be implicated for routine use. Precise body composition determination in NI children leads to an appropriate nutrition intervention to diminish malnutrition. Further studies to validate this new predictive equation with standard methods, compare anthropometric parameters in NI children with different types of etiologies, and determine body composition in NI children with lower severity are needed.

## Conclusion

Malnutrition is common among severe NI children with home enteral nutrition, both undernutrition and overweight. Our study found that FM determined using DDT was associated with age, BMI, SSF and BSF, and we proposed a predictive equation for FM that should be further validated in clinical practice. Assessment of FM together with routine anthropometric measurements provides more effective nutrition monitoring and guides appropriate nutrition support for children with NI.

## Data availability statement

The raw data supporting the conclusions of this article will be made available by the authors, without undue reservation.

## Ethics statement

The studies involving humans were approved by Human Research Ethic Committee, Faculty of Medicine Ramathibodi Hospital, Mahidol University (Protocol No. was COA. MURA2021/390). The studies were conducted in accordance with the local legislation and institutional requirements. Written informed consent for participation in this study was provided by the participants’ legal guardians/next of kin.

## Author contributions

WS: principal investigator, conceptualization, methodology, data collection and analysis, and writing – original draft. OD: conceptualization, methodology, data analysis, revised and finalized manuscript, and supervision. JP and SS: conceptualization, methodology, and revised manuscript. All authors contributed to the article and approved the submitted version.
